# Editorial: Definition of the immune parameters related to COVID-19 severity

**DOI:** 10.3389/fimmu.2023.1204125

**Published:** 2023-04-27

**Authors:** Camilla Tincati, Rory de Vries, Milos Jesenak, Giulia Marchetti

**Affiliations:** ^1^ Clinic of Infectious Diseases, Department of Health Sciences, Azienda Socio Sanitaria Territoriale (ASST) Santi Paolo e Carlo, University of Milan, Milan, Italy; ^2^ Department of Viroscience, Erasmus Medical Center, Rotterdam, Netherlands; ^3^ Department of Paediatrics, Jessenius Faculty of Medicine, Comenius University in Bratislava, University Hospital in Martin, Martin, Slovakia; ^4^ Department of Clinical Immunology and Allergology, Jessenius Faculty of Medicine, Comenius University in Bratislava, University Hospital in Martin, Martin, Slovakia

**Keywords:** SARS-CoV-2, COVID-19 severity, immunity, T-cells, NK cells, antibodies

In December 2019, a novel betacoronavirus was detected in China, identified as severe acute respiratory syndrome coronavirus-2 (SARS-CoV-2), and causally linked to a cluster of cases of severe interstitial pneumonia (1). In March 2020, the coronavirus diseases-2019 (COVID-19) struck the world and has caused, to date, over 760 million cases and almost 9 million deaths (2). The outbreak highlighted the lack of pandemic preparedness globally, yet researchers and physicians all over the world strived to gather knowledge on the pathogenesis of the new disease. A highlight in the control of the pandemic was the rapid development of multiple vaccines based on different platforms, of which over 13.3 billion doses have been administered globally as of April 7, 2023. The present *Research Topic* includes 44 articles on the immune correlates of COVID-19 severity and associated complications.

A solid bulk of evidence in literature highlighted a strong association between hypercytokinemia (also known as “cytokine storm”) and respiratory insufficiency in severe COVID-19 (3). Stemming from these observations, given the dramatic outcome of COVID-19 pneumonia, various research groups investigated whether certain immune parameters could be used as prognostic factors to identify subjects at greatest risk of severe disease. Birindelli et al. developed a laboratory score derived from lymphocyte- and granulocyte-associated parameters by retrospective analysis of 1,619 blood cell counts from 226 hospitalized COVID-19 patients. The score was validated on a new cohort of 140 consecutive COVID-19 patients: a best cut-off score was derived, associated to an overall 82.0% sensitivity and 82.5% specificity for detecting outcome. The scoring trend effectively separated survivor and non-survivor groups. Similarly, Sarif et al. showed that in individuals with acute respiratory distress syndrome (ARDS), the plasma soluble urokinase-type plasminogen activator receptor (suPAR) level was linked to a characteristic plasma proteome, associated with coagulation disorders and complement activation. Importantly, a cut-off value of suPAR was able to predict mortality.

Sánchez-Montalvá et al. conducted a study on 2,600 COVID-19 patients of the first pandemic wave and reported that laboratory tests (*e.g.* neutrophil-to-lymphocyte ratio, C-reactive protein, aspartate, and alanine aminotransferase) were limited predictors due to redundancy; however, the additional use of immunological tests with independent predictive power (CXCL10, IL-6, IL-1RA, and CCL2) could overcome this limitation. Along these lines, Gibellini et al. quantified 62 cytokines and chemokines, as well as other factors involved in inflammation/immunity, in plasma samples collected at hospital admission from 80 hospitalized patients with severe COVID-19, who were stratified on the basis of clinical outcome. The authors found that neutrophilia, lymphocytopenia, procalcitonin, D-dimer and lactate dehydrogenase were strongly associated with the risk of fatal COVID-19. Also Th2 cytokines, markers of cell metabolism and interferons were predictive of life-threatening COVID-19. Villar et al., through a quantitative proteomics approach and multiple data analysis algorithms in 5 patient cohorts, corroborated the predictive value of selected immune-related biomarkers for disease severity, symptomatology and recovery. Accordingly, Kleymenov et al. comprehensively analysed 46 cytokines in the peripheral blood of a large cohort of COVID-19 patients (n=444) and identified TNF-α, IL-10, MIG, IL-6, IP-10, M-CSF, G-CSF, GM-CSF, IFN-α2 as predictors of ICU admission at 4-6 days from symptom onset. In a literature review by Karimi et al., the authors reported that most of the studied markers are able to predict COVID-19 prognosis with neutrophil to lymphocyte ratio (NLR) retaining the greatest prognosticating ability.

Peripheral markers of inflammation were also investigated as predictive factors in clinical settings other than COVID-19 respiratory distress. In particular, Guasp et al. showed that patients with COVID-19-associated encephalopathy and encephalitis presented elevated levels of IL-18, IL-6, and IL-8 in serum and CSF. Guo et al. studied the effects of SARS-CoV-2 infection in placental cells demonstrating that the virus induces pro-inflammatory cytokine and chemokine release, which may contribute to the cytokine storm observed in severely infected pregnant women and related placental dysfunction. Along these lines, SenGupta et al. demonstrated that individuals with type 2 diabetes mellitus displayed higher inflammatory markers and a dysregulated anti-viral and anti-inflammatory response when compared to controls. Finally, Lund Berven et al. studied associations between inflammatory markers, clinical symptoms, pulmonary function and background variables in COVID-19 non-hospitalized patients aged 12 – 25 years and showed alterations of the plasma inflammatory signature in the subacute stage of the infection, despite normal pulmonary functions.

Overall, these studies confirm a pivotal role of the cytokine storm in the pathogenesis of severe COVID-19 and suggest that some markers may be used to identify individuals at greatest risk of disease progression. However, as reviewed by Fouladseresht et al., the prognostic ability of such biomarkers may be impaired by the simultaneous presence of other inflammatory diseases. However, with the ultimate goal of defining tailored therapeutic interventions according to disease severity, the underlying mechanisms causing the cytokine storm should not be overlooked. In this respect, Bigdelou et al. reviewed the molecular implications of concurring diseases in COVID-19 clinical outcome and Premeaux et al. the role of caspases in dictating disease severity. Suhre et al. combined the ‘Olink’ proteomics profiles of newly-recruited and previously published COVID-19 studies and showed protein overexpression in COVID-19 patients with pathways related to cytokine-cytokine interaction, IL-18 signalling, fluid shear stress and rheumatoid arthritis. Qiu et al. performed RNAseq of 126 samples from the GEO database and demonstrated remarkably higher m6A modification levels of blood leukocytes in patients with COVID-19 compared to controls. Similarly, Tang et al., through transcriptome data of blood leukocytes, divided COVID-19 patients into two clusters according to the expression of 35 pyroptosis-related genes and showed that PYRcluster1 patients were in a hyperinflammatory state and had a worse prognosis than PYRcluster2 patients. The hyperinflammation of PYRcluster1 was validated by the results of gene set enrichment analysis (GSEA) of proteomic data.

Overall, these studies push the boundaries of existing knowledge on the pathways underlying the cytokine storm in severe COVID-19 and allow for the identification of possible therapeutic targets. In this view, Marocco et al. demonstrated that tocilizumab can down-regulate sCD163 plasma levels, which were found elevated in COVID-19 pneumonia, while in an *Hypothesis and Theory* article Aloul et al. hypothesized that upregulation of LL-37 could act therapeutically, facilitating efficient NET clearance by macrophages and speeding endothelial repair after inflammatory tissue damage. Novel therapeutic interventions are needed in clinical settings at greatest risk of progression, *i.e.* adults infected with SARS-CoV-2 whilst already hospitalised are at greater risk of mortality compared to those admitted following community-acquired infection, as systematically reviewed by Ponsford et al.

A considerable number of articles in the *Research Topic* assessed the possible interplay of cellular immunity in the pathogenesis of severe COVID-19. Through an integrative stochastic non-linear predictive model of COVID-19 outcome, Elemam et al. demonstrated consistent elevation of IL-15 and IL-10 in severe cases, which are, respectively, stimulators of NK cells and enhancers of NK cell cytotoxicity, denoting a potential critical role of this axis in the COVID-related cytokine storm and following immune-mediated pathologies. The role of NK cells in COVID-19 pathogenesis and their therapeutic implications was comprehensively reviewed by Di Vito et al., while Bobcakova et al. contributed with original findings of lower proportions of NKG2A^+^ NK cells on admission in non-survivors. In the same paper, the authors assessed the role of T cells in COVID-19 pathogenesis, demonstrating the association of higher CD8^+^CD38^+^ cells with fatal outcome. Similar findings were also reported by Du et al. who showed that HLA-DR^+^CD38^+high^CD8^+^ T cells were correlated with COVID-19 disease severity. Furthermore, Clavarino et al. conducted a deep flow cytometry analysis of lymphocyte populations in hospitalized COVID-19 patients and reported that profound CD8^+^ T cell lymphopenia, high levels of CD4^+^ and CD8^+^ T cell activation as well as CD8^+^ T cell senescence were linked to mortality. The data by Al-Attiyah et al. resulted in the definition of a specific immune profile in patients with severe and moderate COVID-19, which was compared to both unvaccinated and vaccinated people in Kuwait. Lower T and B cell levels were shown in these patient cohorts, with significantly higher CD16^+^CD56^+^ NK cells and CD14^+^HLA-DR^+^ monocytes, at the disadvantage of inflammatory CD14^+^CD16^+^HLA-DR^+^ and non-classical CD16^+^HLA-DR^+^ monocytes. These results are in line with those by Legebeke et al., who demonstrated that a transcript signature featuring immunoglobulins, nucleosome assembly, cytokine production and T cell activation, was able to stratify the likelihood of COVID-19 survival.

In a study conducted on *post-mortem* tissues from uninfected and fatal COVID-19 cases, Valdebenito et al. shed light on the possible correlates of peripheral immune dysfunction and lung damage. The authors reported loss of alveolar wall integrity, detachment of large lung tissue pieces, fibroblast proliferation, and extensive fibrosis which were linked to limited CD3^+^CD8^+^ T cell presence, suggesting an exhausted or compromised immune cellular response. Accordingly, Viurcos-Sanabria et al. and Al-Mterin et al. reported, respectively, high PD-1 expression in T cells and upregulation of several immune checkpoint receptors and ligands in COVID-19 patients with severe disease, while Ruiz et al. showed, in a similar setting, low lung IL-1β levels as well as persistence of non-SARS-CoV-2 neutralizing mucosal IgA despite viral clearance.

While all these data point to specific perturbations in both innate and adaptive immune cell subsets in the peripheral blood of COVID-19 patients, some discrepancies exist in the studies, resulting from several differences with regards to the variability of the patient populations. An interesting approach is the one by Jiménez-Cortegana et al., who were interested in evaluating myeloid-derived suppressive cells (MDSCs), given their immunosuppressive role in other disease models. The novelty of this research lies in the search for possible correlations between these cell subtypes and hard outcomes, *i.e.* mortality or survival after ICU admittance. Amongst all the cell populations investigated, a rising trend in granulocytic MDSCs (G-MDSCs) was associated with death, pointing to the possible exploitation of this cell subsets as clinical prognostic factor. Through single cell RNAseq, Li et al. demonstrated that patients recovering from severe COVID-19 showed a circulating immune phenotype different from those recovering from milder disease, therefore demonstrating the persistence of unique immune signatures even after the resolution of acute disease. A comprehensive review on the immune correlates after SARS-CoV-2 infection was conducted by Soleimanian et al.

When considering the possible role of human leukocyte antigen (HLA) genes and COVID-19 susceptibility and severity, two interesting papers were published. In the first one, Ghasemi Basir et al. demonstrated the more essential role of HLA-A versus HLA-B and -C in dictating the immune response to SARS-CoV-2. Interestingly, Mocci et al. presented data from Sardinia, an Italian region struck by the lowest incidence of severe COVID-19, while featuring a high frequency of the Neanderthal risk locus variant on chromosome 3 (rs35044562) that had been considered causative of severe COVID-19. By showing a significant 5-time increased risk of severe disease in Sardinian patients carrying the rs35044562 variant [OR 5.32 (95% CI 2.53 - 12.01), p = 0.000], *vis-a-vis* a 15-time protective effect of the HLA-A*02:01, B*18:01, DRB*03:01 three-loci extended haplotype in the same population [OR 15.47 (95% CI 5.8 – 41.0), p < 0.0001], the authors elegantly demonstrated the existence of a balance between risk and protective immunogenetic factors in this geographic region.

Finally, humoral immunity was studied to understand the role of SARS-CoV-2-specific antibodies in the development of COVID-19. Miyara et al. demonstrated that pre-existing immunity to common cold human coronaviruses can be responsible for recall-type IgG responses to SARS-CoV-2, yet does not lead to cross-protection against SARS-CoV-2 infection. Indeed, neutralizing antibody levels were shown by Macioła et al. to be significantly higher in severe COVID-19 patients, and correlated with both Spike and Receptor Binding Domain (RBD) recognition. In contrast, Hendriks et al. showed that, while a positive association was seen between disease severity and IgG antibody levels, the binding strength decreased with increasing disease severity. Importantly, Cantoni et al. demonstrated a significant reduction in the ability of convalescent sera from the first wave to cross-neutralise following antigenically distinct variants. These findings are in accordance with those by Kurahashi et al., reporting rapid decreases of neutralizing antibodies with a specific epitope for a variant, yet persistence of neutralizing antibodies recognizing the common epitope for several variants.

SARS-CoV-2-specific antibodies were also investigated as possible predictive markers of disease severity. In this respect, Kurano et al. demonstrated that antibody testing may indeed contribute to prediction of the disease maximum severity in COVID-19 patients through analysis models constructed using a machine learning technique. Martynova et al. also shed light on the use of SARS-CoV-2-specific antibodies for diagnostic use, through the inclusion of IgM antibody reactivity with Spike and Nucleocapsid peptides and selected cytokines in a panel specific to patients with a higher risk of fatal COVID-19. Interestingly, Yu et al. showed that IgA detected in saliva could serve as a useful tool for early detection of COVID-19.

The present research topic includes a large number of papers on the correlates of COVID-19 severity that contribute to the current knowledge of pathogenesis. These findings will aid scientific and clinical communities in the development of novel therapeutic and prevention approaches for COVID-19. Moreover, the identified immunologic biomarkers and immune profile changes could serve as an inspiration for the research in the fields of other emerging infectious pathogens.

## Author contributions

CT and GM wrote the manuscript; RV and MJ edited the manuscript. All authors contributed to the article and approved the submitted version.
